# Cochineal for Hooping Cough

**Published:** 1851-06

**Authors:** 


*Cochineal for Hooping Cough.*—An anonymous writer, in the N. Y. Medical Gazette, recommends very highly the following prescription for hooping cough—to be given in teaspoonful doses, three times a day. He regards the cochineal as the active principle of the prescription, and hence gives it in larger doses than usual.

| | |
|---|---|
| Cochineal, in very fine powder, - - | ʒij |
| Carbonate of Potash, - - - - - | ʒj |
| Sugar, - - - - - - - - | ℥j |
| Tincture of Spear-mint, - - - - - | ℥ij |
| Water, - - - - - - - | ℥xiv.—Mix. |

